# Application of sulfur and nitrogen doped carbon quantum dots as sensitive fluorescent nanosensors for the determination of saxagliptin and gliclazide

**DOI:** 10.1098/rsos.220285

**Published:** 2022-06-01

**Authors:** Galal Magdy, Amira A. Al-enna, Fathalla Belal, Ramadan A. El-Domany, Ahmed M. Abdel-Megied

**Affiliations:** ^1^ Department of Pharmaceutical Analytical Chemistry, Faculty of Pharmacy, Kafrelsheikh University, Kafrelsheikh, P.O. Box 33511, Egypt; ^2^ Department of Pharmaceutical Analytical Chemistry, Faculty of Pharmacy, Mansoura University, Mansoura, P.O. Box 35516, Egypt; ^3^ Department of Microbiology and Immunology, Faculty of Pharmacy, Kafrelsheikh University, Kafrelsheikh, P.O. Box 33511, Egypt; ^4^ Department of Pharmaceutical Sciences, Notre Dame of Maryland University, School of Pharmacy, Baltimore, MD 21210, USA

**Keywords:** gliclazide, saxagliptin, sulfur and nitrogen–carbon quantum dots, fluorescent nanosensors, tablets

## Abstract

In this study, highly fluorescent sulfur and nitrogen doped carbon quantum dots (S,N-CQDs) were used as fluorescent nanosensors for direct spectrofluorimetric estimation of each of gliclazide (GLZ) and saxagliptin (SXG) without any pre-derivatization steps for the first time. S,N-CQDs were synthesized employing a simple hydrothermal technique using citric acid and thiosemicarbazide. The produced S,N-CQDs were characterized using different techniques including fluorescence emission spectroscopy, UV spectrophotometry, high-resolution transmission electron microscopy and FT-IR spectroscopy. Following excitation at 360 nm, S,N-CQDs exhibited a strong emission peak at 430 nm. The native fluorescence of S,N-CQDs was quantitatively enhanced by addition of increased concentrations of the studied drugs. The fluorescence enhancement of S,N-CQDs and the concentrations of the studied drugs revealed a wide linear relationship in the range of 30.0–500.0 µM and 75.0–600.0 µM with limits of detection of 5.0 and 10.15 µM for GLZ and SXG, respectively. The proposed method was efficiently used for determination of cited drugs in their commercial tablets with % recoveries ranging from 98.6% to 101.2% and low % relative standard deviation values (less than 2%). The mechanism of interaction between S,N-CQDs and the two drugs was studied. Validation of the proposed method was carried out in accordance with International Conference on Harmonization (ICH) guidelines.

## 1. Introduction

**Figure 1.**
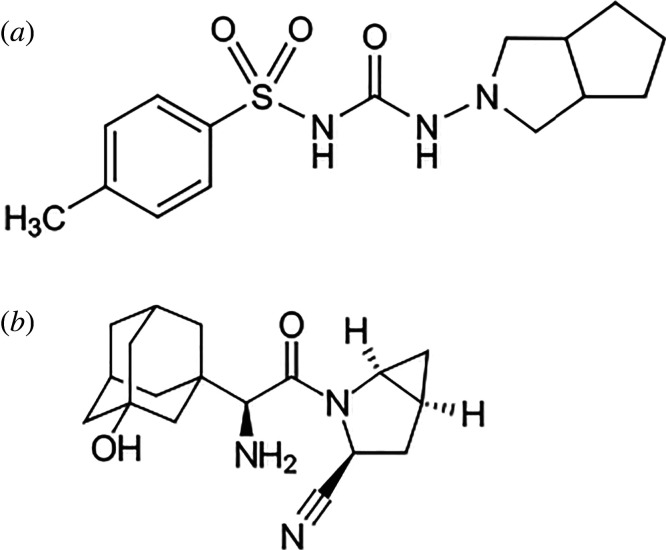
Structural formulae of GLZ (*a*) and SXG (*b*).

Gliclazide (GLZ) (N-[[(hexahydrocylopenta[c]pyrrol-2(1H)-yl)amino]carbony]-4-methylbenzene sulfonamide) (figure 1*a*) is a second generation sulfonylurea used for the control of type 2 diabetes mellitus (DM) by increasing insulin amount secreted by the pancreatic cells [1]. Previous reports showed that GLZ slows down the progression of diabetic retinopathy. It also has low incidence of hypoglycaemia and good tolerability. Consequently, it is considered as the drug of choice for long-term management of non-insulin-dependent DM patients [1,2].

Saxagliptin (SXG) ((1S,3S,5S)-2-[(2S)-2-amino-2-(3-hydroxy-1-adamantyl)acetyl]-2-azabicyclo[3.1.0]hexane-3-carbonitrile) [3] (figure 1*b*) is an oral dipeptidylpeptidase-4 inhibitor (DPP-4) which is considered as a novel therapeutic strategy for type 2 DM. It prevents the inactivation of incretins and also stimulates the glucose dependent insulin release [4].

Different methods for estimating GLZ in dosage forms have been reported. These methods include high performance liquid chromatography (HPLC) [5–7], spectrophotometry [2,8–12] and spectrofluorimetry [13–15]. Similarly, SXG was determined using HPLC [16–18], spectrophotometric [19–24] and spectrofluourimetric [20,22,25] methods. The reported spectrofluorimetric methods for GLZ [13–15] and SXG [20,22,25] required prior chemical derivatization reactions using specific reagents; which in turn renders them time consuming and tedious, with a decrease of their greenness profiles. Whereas, in the current study, a green, rapid and simple spectrofluorimetric method was developed for direct estimation of both GLZ and SXG without the need for pre-derivatization steps for the first time. The proposed approach is based on the quantitative enhancement of the sulfur and nitrogen doped carbon quantum dots (S,N-CQDs) native fluorescence upon increasing the concentrations of the studied drugs.

Carbon quantum dots (CDs) are considered as a novel type of fluorescence nanomaterials that range in size from 2.0 to 10.0 nm. They are biocompatible, non-toxic, easily synthesized from cheap starting materials and chemically stable with good photoluminescence properties. Furthermore, they can be easily functionalized using a variety of species and they are highly water soluble [26–29]. For preparation of fluorescent CDs, various approaches have been reported like chemical oxidation [30], microwave-assisted [31], carbonizing organic methods [32] and hydrothermal synthesis [33]. Synthesis of doped CDs is usually carried out by inserting heteroatoms (such as nitrogen (N), phosphorus (P), sulfur (S), fluorine (F), boron (B)) into the CDs' general structure to enhance their photoluminescence properties [26,34–36]. From literature, CDs were efficiently applied as sensitive probes for the determination of many pharmaceutical compounds in different matrices [26,37,38].

In the present study, a green, simple and economic method was adopted for preparation of S,N-CQDs applying a hydrothermal synthetic approach using thiosemicarbazide (TS) as nitrogen and sulfur source and citric acid (CA) as carbon source producing fluorescence probes for the estimation of each of GLZ and SXG [26].

## 2. Experimental

### 2.1. Materials and reagents

GLZ and SXG were obtained from National Organization of Drug Control and Research Center (NODCAR), Cairo, Egypt. Diamicron Tablets (30 mg GLZ/tablet, batch no. (10)29309, Servier Pharmaceutical Co., Cairo, Egypt) and Formigliptin Tablets (5 mg SXG/tablet, batch no. 001087, Multicare Pharmaceutical Co., Cairo, Egypt) were obtained from a local pharmacy in the Egyptian market. CA, TS, sodium acetate, boric acid, glacial acetic acid, methanol and sodium hydroxide were obtained from Sigma Aldrich (St Louis, MO, USA). Analytical grade chemicals and reagents were used. Double distilled water was used during the study.

Different buffers including borate and acetate buffers (0.2 M) were prepared according to the United States Pharmacopeia (USP) [39].

### 2.2. Instruments and software

UV-1601 PC spectrophotometer was used to carry out UV spectrophotometric measurements (Shimadzu, Kyoto, Japan) using a 1 cm quartz cell. Agilent Technologies' Cary Eclipse fluorescence spectrophotometer was used for fluorescence measurements (Santa Clara, USA). The slit width was adjusted to 5 nm and the instrument was set to 750 V mode. Thermo-Fisher Scientific Nicolet—iS10 FT-IR spectrometer was used to obtain the FT-IR spectra (Waltham, MA, USA). It had a 4000–1000 cm^−1^ deuterated triglycine sulfate (DTGS) detector and a Ge/KBr beam splitter. The measurements were taken in 32 scans with a resolution of 4 cm^−1^. A JEM-2100 high-resolution transmission electron microscope (HRTEM) (JEOL, Tokyo) working at 200 kV was used to investigate morphology of S,N-CQDs. pH-meter (Consort, NVP- 901, Belgium) was also used.

### 2.3. Standard stock solutions

GLZ and SXG are relatively insoluble in water so their standard stock solutions (1.0 mM) were prepared in methanol and different concentrations were obtained by serial dilution with double distilled water as appropriate. The prepared solutions were stable for about 10 days when stored at 4°C.

### 2.4. Synthesis of S,N-CQDs

Synthesis of S,N-CQDs was performed by applying a hydrothermal methodology that was recently reported by Magdy *et al.* [26]. S,N-CQDs were prepared through mixing of 0.52 g CA and 0.68 g TS with 20 ml of double distilled water, and ultrasonication was carried out for 20 min. The mixture was refluxed at 160°C for 12 h until highly fluorescent S,N-CQDs (dark orange colour) were formed, then cooled and kept in the refrigerator for further use.

### 2.5. Fluorescence emission spectroscopy

For GLZ, serial concentrations (0, 30.0, 40.0, 50.0, 100.0, 150.0, 200.0, 300.0, 400.0, 500.0 µM) were mixed with 100 µl of S,N-CQDs at pH 7 using 1 ml of borate buffer, and the mixture was heated at 40°C for 10 min. While for SXG, serial concentrations (0, 75.0, 100.0, 150.0, 200.0, 300.0, 400.0, 500.0, 600.0 µM) were added to 150 µl of S,N-CQDs at pH 11 using 1 ml of borate buffer at 25°C for 10 min. Measurement of the fluorescence intensities for both compounds was carried out at 430 nm following excitation at 360 nm. Calibration curves were constructed by graphing each of GLZ and SXG concentrations (in µM) against the difference in fluorescence intensities, then the corresponding regression equations were generated.

### 2.6. Quantum yield measurements

The following equation [40,41] was used to calculate the quantum yield of S,N-CQDs:

$$\Phi_{S,N-\mathrm{CQDs}}=\Phi_{\mathrm{QS}}\times \left(\frac{F_{S,N-\mathrm{CQDs}}}{F_{\mathrm{QS}}}\;\right)\times {\left(\frac{\eta_{S,N-\mathrm{CQDs}}}{\eta_{\mathrm{QS}}}\right)}^{2}\times \left(\frac{A_{\mathrm{QS}}}{A_{S,N-\mathrm{CQDs}}}\right).$$

where *Φ* is the quantum yield, *F* represents the integrated measured emission intensity, *η* is the solvent refractive index and *A* is the absorbance.

The standard used was quinine sulfate (QS). It was dissolved in 0.1 M H_2_SO_4_ (QY: 0.54 at 350 nm). In the aqueous solutions *η*_S,N-CQDs_/*η*_st_ equals to 1.

### 2.7. Analysis of GLZ and SXG in their tablets

Ten tablets of each of Diamicron or Formigliptin were separately weighed and homogeneously ground. An accurately weighed quantity of the powder corresponding to 30 mg of GLZ or 5 mg of SXG was transferred into a measuring flask (100 ml), followed by addition of 40 ml of methanol. Sonication for 20 min, dilution with methanol to the mark then filtration were performed. Suitable aliquots were transferred from the filtrate into 10 ml measuring flasks, and then the procedure described in §2.5 was performed. The nominal content of tablets was calculated using the corresponding regression equation.

## 3. Results and discussion

### 3.1. Characterization of S,N-CQDs

A facile approach was applied in this study to prepare highly fluorescent S,N-CQDs. The adopted approach is based on the hydrothermal treatment of TS as nitrogen and sulfur source and CA as a carbon source [26]. Under UV light, the S,N-CQDs solution exhibited strong blue fluorescence with a long-lasting homogeneous phase and no apparent precipitation for around 14 days in the refrigerator. Spectrofluorimetry, UV absorption spectroscopy, FT-IR and HRTEM were used to characterize S,N-CQDs.

**Figure 2.**
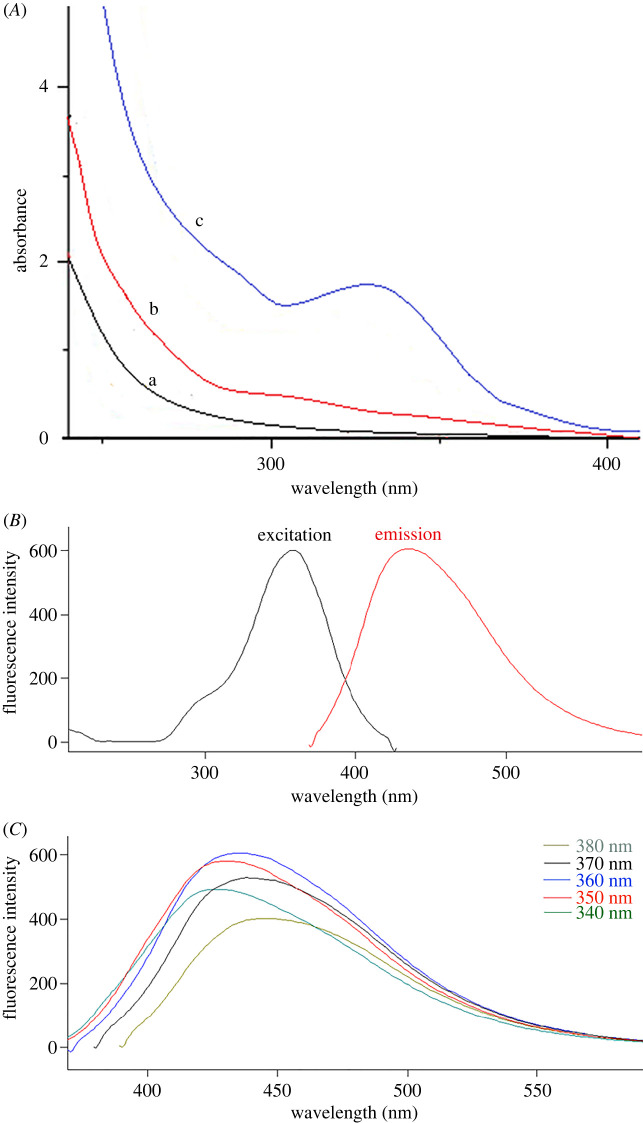
(*A*) UV absorption spectra of citric acid (a), thiosemcarbazide (b) and S,N-CQDs (c); (*B*) fluorescence excitation and emission spectra of S,N-CQDs; (*C*) fluorescence spectra of S,N-CQDs at different excitation wavelengths.

Figure 2*A* shows the UV absorption spectra of S,N-CQDs, CA and TS. S,N-CQDs had a clear UV absorption band at a maximum wavelength of 330 nm [26,42,43]. Figure 2*B* also shows the S,N-CQDs fluorescence emission and excitation spectra in aqueous solution. The optimum excitation and emission wavelengths were found to be 360 and 430 nm, respectively. When the excitation wavelength was changed from 340 to 380 nm, the fluorescence spectra of S,N-CQDs shift, and the highest fluorescence intensity was found at 360 nm (figure 2*C*).

**Figure 3.**
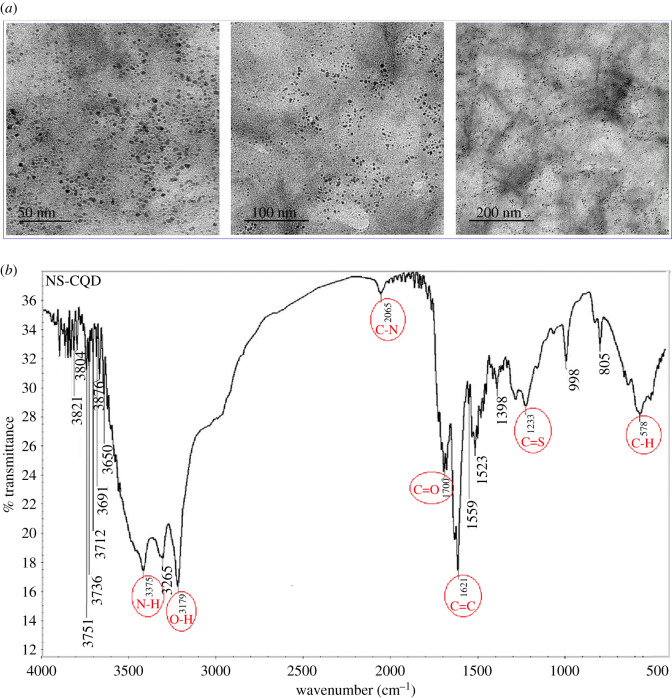
(*a*) The typical HRTEM images of S,N-CQDs; (*b*) FT-IR spectra of N,S-CQDs.

The size and surface morphological characteristics of S,N-CQDs were studied by HRTEM. The samples were placed onto carbon-coated Cu-grid (200 mesh) and investigated using HRTEM at a voltage of 200 kV. As presented in figure 3*a*, S,N-CQDs are spherical in shape and range in size from 8 to 20 nm.

To study the main surface functional groups of S,N-CQDs, FT-IR analysis was used. As shown in figure 3*b*, several distinct vibrational modes were observed. The stretching modes of O–H/N–H groups are represented by broad bands at 3500–3100 cm^−1^. C–N vibration is responsible for the band at 2065 cm^−1^. At 1700 cm^–1^, the C=O of the carboxylic acid group is displayed. C=S and C=C are responsible for the stretching maxima of 1233 and 1621 cm^−1^, respectively. A vibration peak was also observed at 578 cm^−1^ for C–H bond [26,44,45].

The quantum yield of S,N-CQDs was also studied as mentioned in §2.6, and they showed a high quantum yield (58.5%) using QS as a reference.

### 3.2. Interaction mechanism between S,N-CQD and GLZ or SXG

**Figure 4.**
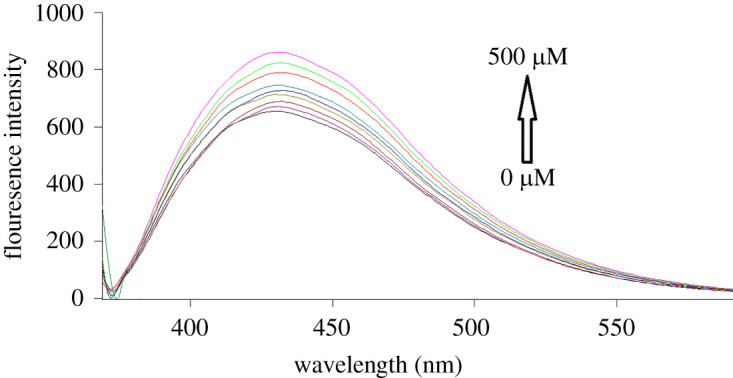
Fluorescence emission spectra of S,N-CQDs in aqueous solution upon addition of various concentrations of GLZ (from bottom to top: 0, 30.0, 40.0, 100.0, 150.0, 200.0, 300.0, 400.0, 500.0 µM).

**Figure 5.**
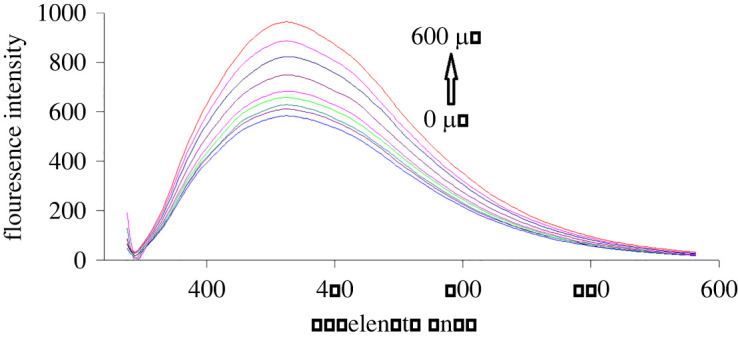
Fluorescence emission spectra of S,N-CQDs in aqueous solution upon addition of various concentrations of SXG (from bottom to top: 0, 75.0, 100.0, 150.0, 200.0, 300.0, 400.0, 500.0, 600.0 µM).

S,N-CQDs emission fluorescence spectra in the presence of various GLZ and SXG concentrations are shown in figures 4 and 5, respectively. By increasing the concentrations of the studied drugs, S,N-CQDs fluorescence intensity was quantitatively enhanced. The native fluorescence of S,N-CQDs was enhanced by 37% and 70% by addition of GLZ (500.0 µM) and SXG (600.0 µM), respectively. The fluorescence enhancement may be attributed to the interaction of each of GLZ and SXG with S,N-CQDs. However, GLZ and SXG cannot form any aggregates with S,N-CQDs, otherwise, the interaction of GLZ or SXG with S,N-CQDs would lead to decrease of surface defects [46–48]. Moreover, the enhancement of fluorescence intensities may be due to saturation of the dangling bonds at the surface of QDs which effectively removes the QDs surface defects. Due to efficient blocking of non-radiative electron/hole recombination on the surface of QDs, the removal of local trap positions leads to formation of more radiative centres. It seems that the increasing concentrations of GLZ and SXG in the solution get attached to the QDs surface and thereby correct the defective energy levels. This removal of defect levels improves the exciton emissions and subsequently enhances the fluorescence emission intensities of the QDs [46–48].

### 3.3. Inner filter effect of GLZ and SXG

**Figure 6.**
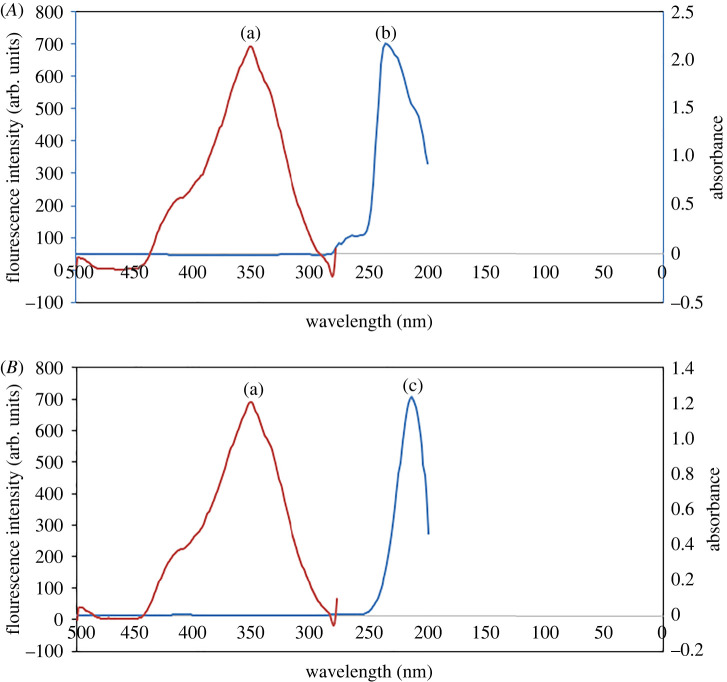
(*A*) A co-plot showing that there is no overlap between fluorescence excitation spectrum of the S,N-CQDs (a) and the UV–Vis absorption spectrum of the GLZ (500.0 µM) (b); (*B*) A co-plot showing that there is no overlap between fluorescence excitation spectrum of the S,N-CQDs (a) and the UV–Vis absorption spectrum of the SXG (600.0 µM) (c).

The inner filter effect of the studied drugs was carefully studied to confirm that the enhancement of the fluorescence intensity of S,N-CQDs is due to the interaction between QDs and drugs and not due to the native fluorescence of the studied drugs. It was found that 500.0 µM of GLZ and 600.0 µM of SXG (the maximum concentrations in this study) have neither native fluorescence at 430 nm after excitation at 360 nm nor absorbance at 360 nm, indicating that, there was no inner filter effect of either GLZ or SXG (figure 6).

### 3.4. Optimization of factors affecting interaction of GLZ and SXG with S,N-CQDs

To achieve the maximum sensitivity of the method for GLZ and SXG determination, different factors influencing the fluorescence intensities were studied; including pH of the medium, incubation time and temperature.

#### 3.4.1. Effect of pH

**Figure 7.**
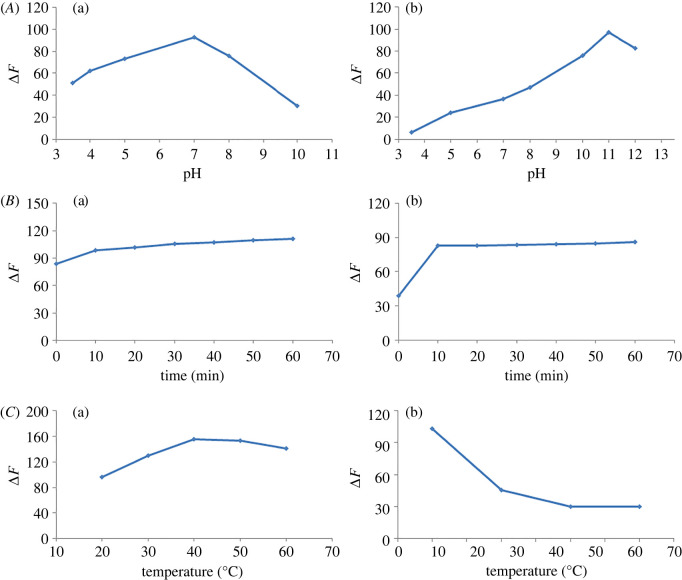
Effect of pH (*A*), incubation time (*B*) and temperature (*C*) on the enhancement of the fluorescence intensity of S,N-CQDs by GLZ (300.0 µM) (a), SXG (200.0 µM) (b).

To investigate the impact of pH, the experiments were carried out over pH range from 3.5 to 12 using acetate and borate buffers. It was found that the maximum S,N-CQDs emission intensities were achieved at pH 7 and 11 for GLZ and SXG, respectively (figure 7*A*). Accordingly, the volume of borate buffer was examined from 0.5 to 4 ml, and it was found that the optimum volume is 1 ml for both drugs.

#### 3.4.2. Incubation time

The incubation time of S,N-CQDs with the investigated drugs was studied by recording the emission fluorescence spectra at time intervals ranging from 1 min to 1 h. The obtained outcomes displayed a fast increasing response after mixing the cited drugs and QDs that reached a constant value after 10 min. The fluorescence signals remained stable for 1 h, giving the suggested method an additional advantage (figure 7*B*).

#### 3.4.3. Effect of temperature

Over a temperature range of 20–60°C, the influence of temperature on signal enhancing effect of GLZ and SXG was studied. The obtained results demonstrated that the maximum response was achieved at 40°C and 25°C for GLZ and SXG, respectively (figure 7*C*).

### 3.5. Method validation

The developed method was validated according to ICHQ2(R1) guidelines [49].

#### 3.5.1. Linearity and range

The calibration plots were constructed by graphing the concentrations of each drug (μM) versus the difference in fluorescence intensity (Δ*F* *= F* − *F*_0_). The linear range was found to be 30.0–500.0 µM and 75.0–600.0 µM for GLZ and SXG, respectively. The regression equations that represent rectilinear relationship are as follows:

3.1 $$F-F_{0}=0.37C+20.83\;(r=0.9999)\;\mathrm{for}\;\mathrm{GLZ}$$

and

3.2 $$F-F_{0}=0.67C+28.90\;\;(r=0.9998)\;\mathrm{for}\;\mathrm{SXG},$$

where *F*_0_ and *F* are the QDs fluorescence intensities in absence and presence of cited drugs, respectively, C is the concentration in μM. Values of correlation coefficients (*r*) approximating unity indicate the acceptable linearity of the developed procedure (table 1).

**Table 1.** Analytical performance data for the proposed method.

parameter	GLZ	SXG
linearity range (µM)	30.0–500.0	75.0–600.0
limit of detection (LOD)^a^ (µM)	5.0	10.16
limit of quantitation (LOQ)^b^ (µM)	15.17	30.78
regression equation	*F − F*_0_ = 0.37C + 20.83	*F − F*_0_ = 0.67C + 28.90
correlation coefficient (*r*)	0.9999	0.9998
standard deviation (s.d.)	1.01	1.87
percentage relative standard deviation (% RSD)	1.01	1.86
*S_y/x_*, s.d. of the residuals	0.95	3.10
*S_a_*, s.d. of the intercept	0.56	2.07
*S_b_*, s.d. of the slope	0.0021	0.0061

^a^LOD = 3.3 *S_a_*/*b*.

^b^LOQ = 10 *S_a_*/*b*, where *S_a_* is the standard deviation of the intercept and *b* is the slope.

#### 3.5.2. LOD and LOQ

The following equations were used to calculate limit of quantitation (LOQ) and limit of detection (LOD) values:

LOD = 3.3 *S_a_*/*b*, LOQ = 10 *S_a_*/*b*, where *S_a_* is the standard deviation of *y*-intercept, *b* is the slope. The obtained results are abridged in table 1.

#### 3.5.3. Precision and accuracy

Three different concentrations of the cited drugs and three replicates of each concentration were used to test the intra-day and inter-day precisions. The obtained results showed small % relative standard deviation (RSD) values (less than 2%), indicating an acceptable precision of the developed approach (table 2). The accuracy and precision were demonstrated by statistical comparison of the obtained results with those given by comparison methods for both drugs [2,23], showing insignificant difference between them as presented by *t* and *F* values, respectively [50] (table 3).

**Table 2.** Intra-day and inter-day precision data for the determination of the studied drugs by the proposed method. Each result is the average of three separate determinations.

analyte	conc. taken (µM)	intra-day^a^			inter-day^b^		
		conc. found ± s.d. (μM)	% RSD	% error^c^	conc. found ± s.d. (μM)	% RSD	% error^c^
GLZ	100.0	101.01 ± 0.93	0.92	0.53	101.11 ± 1.32	1.31	0.75
	200.0	197.69 ± 0.86	0.87	0.5	198.82 ± 1.51	1.52	0.88
	300.0	302.14 ± 0.98	0.97	0.56	301.21 ± 1.49	1.48	0.86
SXG	100.0	101.1 ± 1.1	1.09	0.63	102.07 ± 1.24	1.22	0.7
	200.0	196.09 ± 0.63	0.65	0.37	196.81 ± 0.7	0.71	0.41
	300.0	293.84 ± 0.36	0.37	0.21	294.3 ± 0.73	0.74	0.43

^a^Within the day.

^b^Three consecutive days.

^c^% Error = % RSD/√*n*.

**Table 3.** Application of the proposed method for the determination of GLZ and SXG in pure forms.

parameter	proposed method			
	GLZ		SXG	
	conc. taken (µM)	% found^a^	conc. taken (µM)	% found^a^
	30.0	100.21	75.0	102.94
	40.0	100.18	100.0	102.23
	100.0	100.93	150.0	102.03
	150.0	99.6	200.0	98.17
	200.0	98.15	300.0	98.36
	300.0	101.61	400.0	99.68
	400.0	99.86	500.0	99.08
	500.0	99.82	600.0	101.15
mean ± s.d.		100.05		100.46
		1.01		1.87
% RSD		1.011		1.859
% error		0.357		0.66
	comparison method			
	GLZ [2]		SXG [23]	
mean ± s.d.	100.56 ± 2.48		99.18 ± 2.35	
*N*^c^	3.0		3.0	
*F*-value	5.33 (19)^b^		4.74 (19)^b^	
*t*-value	0.29 (2.77)^b^		0.58 (2.77)^b^	

^a^Mean of three determinations.

^b^The values between parentheses are the tabulated *t*- and *F*-values at *P* = 0.05 [50].

^c^Number of samples.

#### 3.5.4. Robustness

The method robustness was studied by examining the influence of slight variations in experimental parameters affecting fluorescence intensity of studied drugs. The influence of incubation time (10 ± 1 min) for both drugs, pH (7 ± 0.1) for GLZ and (11 ± 0.1) for SXG, temperature (40 ± 1°C) for GLZ and (25 ± 1°C) for SXG and S,N-CQDs volume (100.0 µl ± 1) for GLZ and (150.0 µl ± 1) for SXG were investigated and showed insignificant impact on % RSD values and % recoveries (table 4).

**Table 4.** Robustness evaluation of the proposed method.

variation	% recovery	% RSD
GLZ		
1. Volume of N,S-CQDs (100.0 µl ± 1)		
99.0 µl	97.97	1.24
100.0 µl	98.48	1.29
101.0 µl	100.43	1.31
2. Incubation time (10 ± 1 min)		
9 min	101.11	1.09
10 min	100.35	1.06
11 min	102.46	1.07
3. pH (7 ± 0.1)		
6.9	100.89	1.13
7	100.12	1.07
7.1	100.41	1.11
4. Temperature (40 ± 1°C)		
39°C	97.21	1.59
40°C	100.41	1.68
41°C	98.06	1.62
SXG		
1. Volume of N,S-CQDs (150 µl ± 1)		
149 µl	100.05	0.54
150 µl	100.88	0.41
151 µl	100.44	0.63
2. Incubation time (10 ± 1 min)		
9 min	100.74	0.53
10 min	101.78	0.73
11 min	102.18	0.64
3. pH (11 ± 0.1)		
10.9	101.82	0.86
11	100.93	0.8
11.1	100.21	0.79
4. Temperature (25 ± 1°C)		
24°C	99.37	0.73
25°C	100.01	0.64
26°C	100.64	0.61

#### 3.5.5. Selectivity

The proposed method's selectivity was proved by its capacity to estimate the cited drugs in presence of other antidiabetic drugs including metformin, dapagliflozin, empagliflozin, canagliflozin, alogliptin, omarigliptin and glebenclamide. The tolerance limits of these drugs were calculated as the concentration that results in 2% relative error [51] and the obtained results are summarized in table 5. In addition, the method selectivity was confirmed by its capacity to analyse the drugs in their tablets with low % RSD values (less than 2%) and high % recoveries (98.64–101.21%); demonstrating no interference from common excipients (table 6).

**Table 5.** Tolerance limits for other antidiabetic drugs in presence of GLZ and SXG.

drug	tolerance limit with GLZ (µM)	tolerance limit with SXG (µM)
omarigliptin	96	16
alogliptin	13	13
glebenclamide	5	17
metformin	15	10
dapagliflozin	111	55
canagliflozin	90	16
empagliflozin	50	23

**Table 6.** Application of the proposed method for the determination of GLZ and SXG in tablet dosage forms.

GLZ				
pharmaceutical preparation	proposed method		comparison method [2]	
	conc. taken (µM)	% found^a^	conc. taken (µM)	% found^a^
Diamicron tablets (30 mg GLZ/tab)	30.0	99.19	30.0	100.55
	60.0	100.83	60.0	98.73
	90.0	99.73	90.0	99.94
mean ± s.d.		99.92 ± 0.84		99.74 ± 0.93
*F*-value	1.23 (19)^b^			
*t*-value	0.25 (2.77)^b^			
SXG				
	proposed method		comparison method [23]	
pharmaceutical preparation	conc. taken (µM)	% found^a^	conc. taken (µM)	% found^a^
Formigliptin tablets (5 mg SXG/tab)	75.0	101.21	75.0	96.72
	100.0	98.64	100.0	102.54
	150.0	100.30	150.0	98.76
mean ± s.d.		100.05 ± 1.3		99.34 ± 2.95
*F*-value	5.14 (19)^b^			
*t*-value	0.38 (2.77)^b^			

^a^Mean of three determinations.

^b^The values between parentheses are the tabulated *t*- and *F*-values at *p* = 0.05 [50].

### 3.6. Analysis of GLZ and SXG in their tablets

Due to the high selectivity and reproducibility of the developed method, it was efficiently used to estimate the cited drugs in their commercial tablets with low % RSD values and high % recoveries. Statistical analysis of obtained results with those given by comparison methods for GLZ and SXG [2,23], showed insignificant difference between them regarding precision and accuracy as indicated by the *F*- and *t*-values, respectively [50] (table 6).

## 4. Conclusion

In this study, a facile hydrothermal approach was used for synthesis of S,N-CQDs using CA and TS. The prepared S,N-CQDs were characterized using various techniques. S,N-CQDs were used as fluorescent nanosensors for estimation of each of GLZ and SXG depending on their enhancement effect on S,N-CQDs fluorescence intensities. The developed method showed good selectivity for estimation of GLZ and SXG in their tablets and in presence of other antidiabetic drugs. The proposed method is rapid, simple and cost-effective without the need for sophisticated instruments or prior derivatization of the studied drugs. Full validation of the proposed method was performed in accordance with ICH recommendations.

## Data Availability

Data available from the Dryad Digital Repository: https://doi.org/10.5061/dryad.8cz8w9gsm [52].
